# Possible role of human ribonuclease dicer in the regulation of R loops

**DOI:** 10.1002/2211-5463.70026

**Published:** 2025-03-24

**Authors:** Klaudia Wojcik, Paulina Krzeminska, Anna Kurzynska‐Kokorniak

**Affiliations:** ^1^ Department of Ribonucleoprotein Biochemistry Institute of Bioorganic Chemistry Polish Academy of Sciences Poznan Poland

**Keywords:** cancer, G‐quadruplexes, long non‐coding RNAs, R loops, ribonuclease Dicer, TERRA

## Abstract

R loops are three‐stranded nucleic acid structures that form naturally in cells under various conditions, mainly as intermediates during replication or as by‐products during transcription. R loops are involved in the regulation of many important cellular processes, including replication, transcription, centromere stabilization, protection of chromosome ends, or control of telomere length. Unscheduled R loops are linked to many diseases, including cancer, neurodegenerative, or inflammatory disorders. The list of cancer diseases linked to excessive R loop accumulation is growing rapidly. There is currently much debate about the understanding of abnormal R loop formation and its impact on genome instability and cancer development. In this review, we briefly describe the nature of R loops, their formation under physiological and pathological conditions, and the proteins involved in the regulation of R loops. In addition, we emphasize the possible role of the human ribonuclease Dicer, a multi‐tasking protein mostly known for its important role in microRNA biogenesis, in the regulation of R loops. We also discuss the involvement of R loops in cancer development and their potential use as diagnostic biomarkers. Knowledge of the molecular mechanisms underlying R loop dysregulation may significantly improve our understanding of cancer biology and provide new directions for research.

## Graphical abstract

R loops play an important role in regulating key cellular processes such as replication, transcription, centromere stabilization, or control of telomere length. However, the unscheduled accumulation of R loops can cause many diseases, including cancer, and neurodegenerative or inflammatory disorders. Interestingly, accumulating data indicate a possible role of human ribonuclease Dicer in the regulation of R loops.

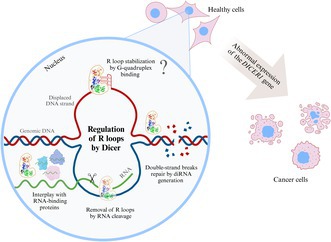

**Abbreviations**
AID: activation‐induced deaminase
ALL: acute lymphoblastic leukemia
ALT: alternative lengthening of telomere
AML: acute myeloid leukemia
CLL: chronic lymphocytic leukemia
CML: chronic myeloid leukemia
CSR: class‐switch recombination
DDX5: DEAD‐box helicase 5
diRNA: damage‐induced small RNA
DNMT: DNA methyltransferase
DSBs: double‐strand breaks
dsDNA: double‐stranded DNA
dsRNA: double‐stranded RNA
eRNA: enhancer RNA
hDicer: human Dicer
HOTTIP: HOXA transcript at the distal tip
HP1γ: heterochromatin protein 1γ
lncRNA: long non‐coding RNA
LSD1: lysine‐specific demethylase 1A
miRNA: microRNA
mtDNA: mitochondrial DNA
RAD51AP1: RAD51‐associated protein 1
rDNA: ribosomal DNA
sEVs: small extracellular vesicles
siRNA: small interfering RNAs
ssDNA: single‐stranded DNA
TAD: topologically associating domains
TERRA: telomeric repeat‐containing RNA
THCA: thyroid cancer
Thrap3: thyroid hormone receptor‐associated protein 3
TRF2: telomeric repeat‐binding factor 2

R loops are three‐stranded nucleic acid structures that consist of a DNA–RNA hybrid and a displaced single‐stranded DNA (ssDNA) [1]. Among the physical properties of chromatin that promote the opening of the double‐stranded DNA (dsDNA) and the formation of an R loop are negative DNA supercoiling, high GC content, and the breakage of chromosomal DNA. In addition, R loops can be stabilized by G‐quadruplex structures (Fig. 1) [2]. R loops form naturally as intermediates during replication or as by‐products during transcription. They also regulate telomere length and protect chromosome ends [3].

**Fig. 1:**
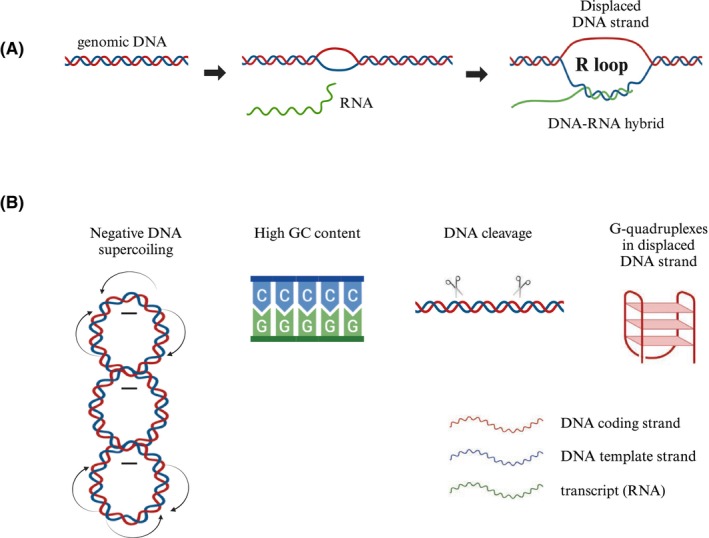
Schematic representation of the R loop formation (A) and factors promoting R loop formation and stabilization (B). Figure generated in BioRender software.

## R loops as natural intermediates and regulators

R loops form during DNA replication initiation in mitochondrial DNA (mtDNA) [4], bacterial plasmids [5], and some bacteriophages [6, 7]. In these cases, RNA produced by the RNA polymerase serves as a primer for replication initiation. Interestingly, R loop formation has been found to regulate the copy number of mtDNA. It is known that a somatic cell contains 1000–5000 mitochondria, each with 5–10 copies of mtDNA [8]. In the case of gametes: spermatozoa contain less than one hundred mitochondria, each with a single mtDNA, and oocytes have about 100 000–400 000 mitochondria, also with a single mtDNA copy [9, 10].

R loops are also involved in immunoglobulin class‐switch recombination (CSR) [11, 12]. CSR is important for the production of different classes of immunoglobulin by B lymphocytes after contact with a pathogen [13]. It is hypothesized that R loops boost mutation rates by generating long tracts of ssDNAs that serve as the substrate for activation‐induced deaminase (AID), the enzyme that co‐transcriptionally mutates ssDNA in so‐called switch recombination sequences [14]. AID‐induced mutations lead to DNA breakage and subsequent repair of two DNA sequences, which ultimately result in class‐switch recombination [15].

R loops can also be formed by long non‐coding RNAs (lncRNAs) [16]. A well‐known example of such structures is a telomeric R loop mediated by a telomeric repeat‐containing RNA (TERRA) [17, 18, 19]. TERRA, a lncRNA transcribed by RNA polymerase II from telomeric DNA sequences present at the ends of eukaryotic chromosomes [20, 21], can invade telomeric DNA through direct base‐pairing to form R loop structures. G‐rich repeats (in humans, TTAGGG), single‐strand breaks, and G‐quadruplex structures in telomere sequences promote telomeric R loop formation. TERRA can regulate the telomeres [22, 23, 24]. The interaction between TERRA and G‐quadruplexes at telomeres plays a critical role in maintaining telomere stability [25]. The shelterin complex, composed of six proteins: TRF1, TRF2, POT1, RAP1, TIN2, and TPP1, serves as the primary regulator of TERRA, playing a crucial role in maintaining telomere stability and protecting chromosome ends [18, 19]. TRF1, TRF2, and POT1 bind directly to telomeric DNA sequences. The remaining three (RAP1, TIN2, and TPP1) are associated with telomeres via protein–protein interactions [18, 19]. The shelterin complex is also involved in the regulation of the R loops [18, 19, 26]. Other examples of lncRNAs that can form R loops are enhancer RNAs (eRNAs) [27, 28]. eRNAs are relatively long non‐coding RNA molecules (50–2000 nucleotides) that are transcribed by RNA polymerase II from the DNA sequence of enhancer regions. eRNAs hybridize with their corresponding DNAs to form R loop structures [29]. In this way, eRNAs may modulate the activity of the matching enhancer in target genes. It has been shown that increased level of eRNAs leads to unscheduled R loop formation and consequent genomic instability at enhancer regions [30], which is causally linked to tumorigenesis.

R loop formation may also induce antisense transcription, leading to double‐stranded RNA (dsRNA) generation, followed by H3K9me2 marks and recruitment of heterochromatin protein 1γ (HP1γ) [31]. These events lead to RNA polymerase II pausing and transcription termination [31]. In addition, R loop formation may protect CpG islands within the promoter regions from DNA methyltransferases (DNMTs), which in consequence affect methylation‐dependent repression of transcription [32].

## R loops as transcriptional by‐products

R loops are primarily transcriptional by‐products and are abundant in nucleolar regions, where RNA polymerase I drives the expression of ribosomal DNA (rDNA) [1]. These sites of R loop formation are particularly critical for genome stability, as RNA polymerase I accounts for more than 60% of total transcription [33].

R loop structures form also at centromeric regions [34]. Centromeres are chromosomal fragments responsible for the proper distribution of DNA during cell division. They consist of repetitive α‐satellite sequences that are intensely transcribed and form DNA–RNA hybrids. R loop structures at centromeres are recognized by and associated with BRCA1, a well‐known tumor suppressor preventing the accumulation of DNA–RNA hybrids [35]. In budding yeast, unscheduled R loops at centromeric regions have been found to contribute to defects in kinetochore biorientation and chromosomal instability [34].

One report has shown the importance of active transcription, the presence of a functional poly(A) signal, and termination G‐rich pause elements in the formation of R loops [36]. It has been observed that depletion of senataxin, a known RNA/DNA helicase, leads to the accumulation of R loops specifically downstream of the poly(A) signal, emphasizing a senataxin's role in resolving these structures in transcription termination regions [36]. This finding suggests that while R loops may facilitate certain aspects of transcription termination, their persistence can be detrimental, highlighting the critical role of senataxin in clearing R loops to ensure proper gene expression and maintain genomic stability [36].

## R loops as potentially harmful structures

R loops may impair replication fork progression and transcription; therefore, their removal is essential to preserve genome integrity [11, 37]. Replication and transcription occur during the cell cycle. Since replication and transcription use the same DNA strand as a template, the cell cycle must be tightly regulated. Conflicts between these two events can result in errors in DNA replication and/or protein synthesis, both of which are essential for normal cell division [38]. R loop formation and removal is an important mechanism regulating these two processes. When replication and transcription proceed in opposite directions, RNA polymerase II cannot function due to a collision with DNA polymerase, and R loops accumulate, whereas co‐directional collision does not result in R loop accumulation [38]. R loops have been found during several stages of cell division: G1, G2, M phases, and mostly in S phase. The S phase leads to DNA synthesis, which requires DNA replication and transcription to produce multiple proteins [39]. R loops can inhibit transcription and enable DNA replication, especially during meiosis [40, 41]. Thus, precise processing of R loops is crucial to maintain the balance between replication and transcription.

Displaced ssDNAs in R loop structures are susceptible to damage. Unscheduled R loops often lead to replication stress, as they can stall replication forks, potentially causing double‐strand breaks (DSBs) [42]. Moreover, unscheduled R loops may disturb the balance between replication and transcription, which may contribute to genomic instability. To counteract this, cells employ a variety of enzymes involved in R loop regulation and resolving, including topoisomerases, RNA/DNA helicases, chromatin modulators, RNA processing factors, and ribonucleases [43, 44]. Examples of such proteins and their roles in the cell are summarized in Table 1.

**Table 1:** Exemplary enzymes involved in the regulation of R loops.

Group	Enzyme	Role	Source
Topoisomerases	Topoisomerase I (TOP1)	Relaxes DNA supercoiling and prevents R loop formation	[118]
RNA/DNA helicases	DEAD‐Box Helicase 1 (DDX1)	Plays a crucial role in the formation of R loops over immunoglobulin heavy‐chain (IgH) switch regions by targeting AID (activation‐induced cytidine deaminase) through a post‐transcriptional mechanism. It binds to G‐quadruplex structures within switch transcripts and facilitates their conversion into R loops in the switch recombination sequences (the S‐regions)	[119]
	Senataxin (SETX)	Catalyzes the unwinding of DNA–RNA hybrid in R loops, promoting their resolution	[120]
Chromatin modulators	Metastasis‐associated protein 2 (MTA2)	Regulates chromatin dynamics in regions where R loops are formed, promoting genome stability by controlling access to DNA	[121]
	DNA‐dependent ADP‐ribosyl transferase (PARP1)	Binds to R loops and initiates DNA repair processes, preventing genomic instability	[122]
	Breast cancer type 1 susceptibility protein (BRCA1)	Prevents accumulation of DNA–RNA hybrids	[35]
RNA processing factors	The conserved THO complex (THOC)	Regulates TERRA‐associated R loops by binding to nucleoplasmic TERRA and reducing R loop accumulation at telomeres, thus maintaining telomere stability	[123]
Ribonucleases	RNase H1	Degrades the RNA portion of the R loops, returning the two DNA strands to dsDNA form	[124]
	RNase H2	Degrades RNA in DNA–RNA hybrids, including RNA primers and ribonucleotides erroneously incorporated into DNA. The activity of RNase H2 is mainly found during cell division, in the G2/M checkpoint. This phase of the cell cycle controls the quality of replicated DNA and allows or prevents cells from dividing	[43, 125]
	RNA Exonuclease 5 (REXO5)	Plays a key role in the physiological control of R loops using its exonuclease domain	[126]
	Dicer	Cleaves the RNA strand of DNA–RNA hybrids within R loop structures	[57]
Recombinase	DNA repair protein (RAD51)	RAD51 interacts with TERRA and catalyzes R loop formation, a direct role in TERRA recruitment via strand invasion	[19, 127]

## Ribonuclease dicer as a multi‐tasking protein

**Fig. 2:**
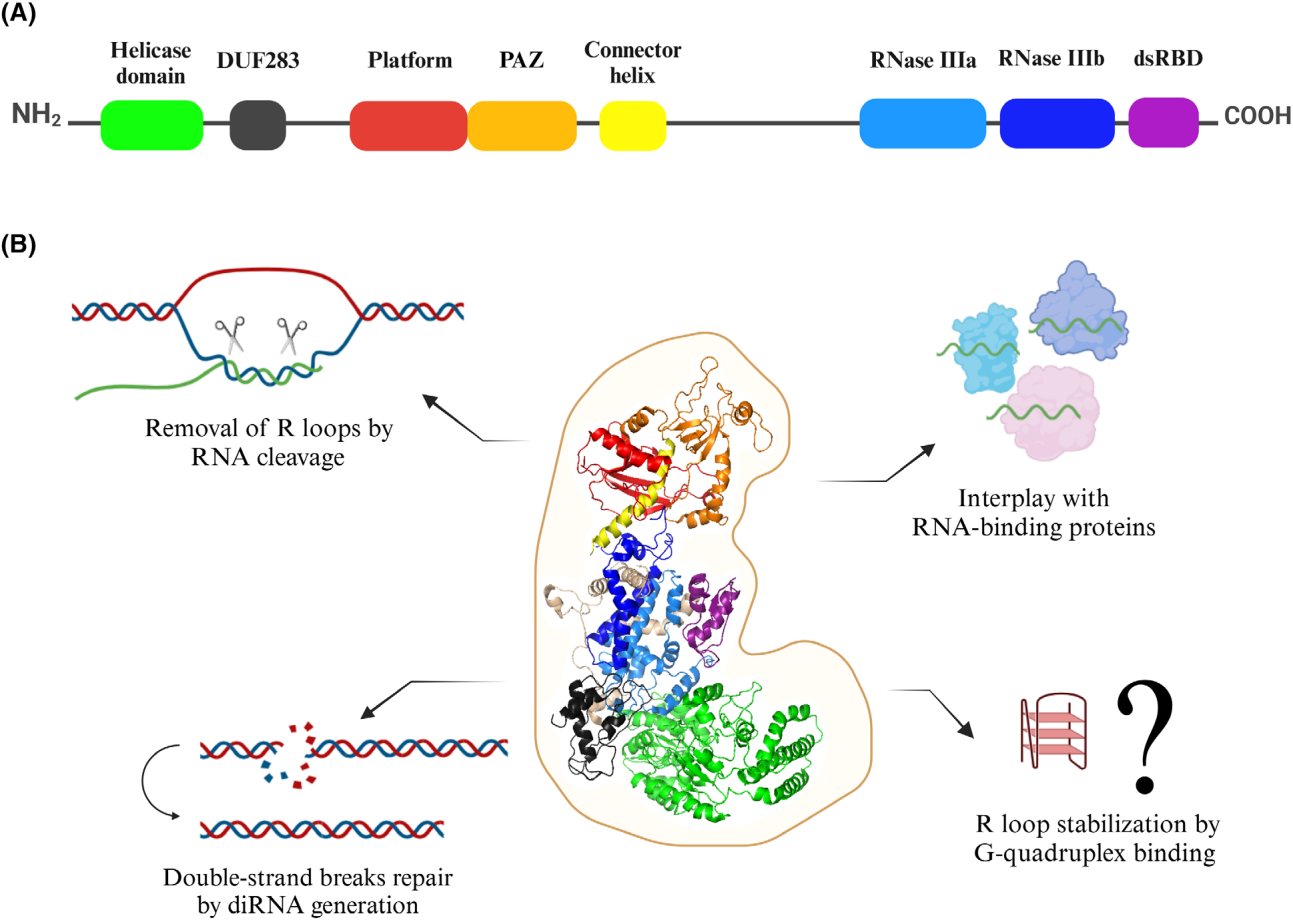
Dicer as a multi‐tasking protein. (A) Schematic showing the domain organization of hDicer. Domains are color‐coded: helicase (green), DUF283 – a domain of unknown function 283 (dark gray), Platform (red), PAZ – Piwi‐Argonaute‐Zwille domain (orange), Connector helix (yellow), RNase IIIa (blue), RNase IIIb (dark blue) and dsRBD—a dsRNA‐binding domain (purple). (B) A proposed Dicer's role in the R loop level regulation. The 3D structure of hDicer (PDB entry 5ZAL) [128] was visualized by PyMOL. The domains are color‐coded as in (A). Figure generated in BioRender software.

The Dicer ribonuclease is mostly known for its important role in the biogenesis of small regulatory RNAs, microRNAs (miRNAs) and small interfering RNAs (siRNAs) [45, 46]. This canonical role of Dicer is associated with its cytoplasmic localization [47]. Dicer can also function in the nucleus. For example, it is already known that nuclear Dicer can participate in chromatin structure remodeling [48, 49, 50, 51, 52], restrict the deleterious accumulation of endogenous dsRNAs [53] or contribute to DNA damage response activation by generating small non‐coding RNAs, referred to as damage‐induced small RNAs (diRNAs) or DDRNAs [54, 55, 56]. Interestingly, a recent study has shown that ribonuclease Dicer may be involved in nuclear R loop processing [57]. Under the *in vitro* conditions and in the cell, human Dicer (hDicer) cleaved RNA within harmful R loops, but not within the DNA–RNA hybrid without a loop, which suggested that hDicer activity was specific for the R loop structures [57]. Silencing of the human *DICER1* gene led to the accumulation of R loop structures in the nuclei, and even the overexpression of other ribonucleases involved in the R loop removal did not counteract the accumulation of R loops in the cells with downregulated *DICER1* expression [57]. It has been suggested that R loop removal by hDicer is supported by its annealing activity through facilitating DNA–RNA hybrid formation [57]. The annealing activity of hDicer was first revealed [55] and extensively characterized [56, 58] in our laboratory. We demonstrated that hDicer can support hybridization between complementary sequences present in nucleic acids even when both of them are trapped within secondary structures [55, 56]. Besides cleaving the RNA within the R loop structures, hDicer might influence R loops by involvement in DNA damage repair. Specifically, hDicer has been implicated in the DNA damage response and repair of DSBs in mammalian cells due to replication stress [59]. As mentioned above, during DSB stress, hDicer produces diRNAs that are essential for the repair of damaged DNA [60, 61]. These small RNAs correspond to the sites of DSBs and are thought to serve as templates for efficient DNA repair [60]. In addition, hDicer can potentially interplay with other R loop‐binding proteins, by direct interactions; e.g., helicase DHX9 [62, 63, 64], or indirectly; e.g., BRCA2/BRCA1 [65, 66], BRD4 [67, 68], SETX [69, 70]. Furthermore, hDicer can potentially stabilize R loops by binding to G‐quadruplexes. Importantly, the results of our recent studies have indicated that hDicer binds both DNA and RNA G‐quadruplexes, including TERRA, with high affinity [71]. The potential of hDicer to interact with G‐quadruplexes and R loops, two structures with regulatory functions in the cell, strongly indicates that this ribonuclease is a multi‐tasking protein not only involved in miRNA and siRNA biogenesis, but also in many other cellular pathways (summarized in Fig. 2).

## Dysregulation of hDicer in cancer

Many cases have been reported in which abnormal *DICER1* expression was correlated with the development of carcinogenesis; reviewed in [46]. In the context of a specific cancer, disturbances in *DICER1* expression should be considered at multiple levels, including mutations in the *DICER1* gene, the level of *DICER1* expression, as well as global miRNA expression. As mentioned above, disturbed levels of hDicer may also affect the R loop accumulation in cells [57]. On the one hand, *DICER1* overexpression can cause excessive R loop removal, either directly (by RNA cleavage) or indirectly (by impaired miRNA biogenesis). On the other hand, decreased levels of hDicer can cause ineffective removal of R loops in cells, which threaten genome integrity [57]. Here, we focus on aberrant *DICER1* expression in leukemia and thyroid cancer.

**Fig. 3:**
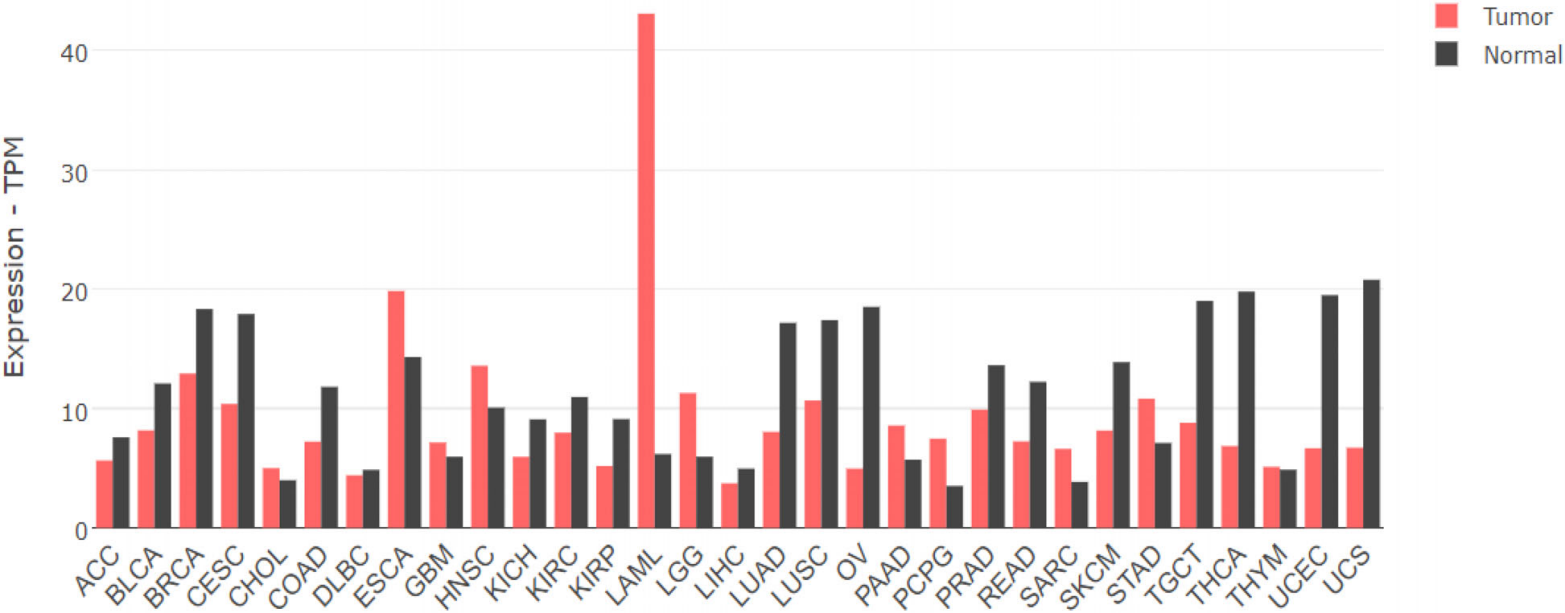
Expression levels of *DICER1* in various cancers; based on the data obtained from the GEPIA2 database, which provides extensive resources for gene expression analysis derived from cancer and normal samples in the TCGA and GTEx databases [73]. ACC, adrenocortical carcinoma; BLCA, bladder urothelial carcinoma; BRCA, breast invasive carcinoma; CESC, cervical squamous cell carcinoma and endocervical adenocarcinoma; CHOL, cholangio carcinoma; COAD, colon adenocarcinoma; DLBC, lymphoid neoplasm diffuse large B‐cell lymphoma; ESCA, esophageal carcinoma; GBM, glioblastoma multiforme; HNSC, head and neck squamous cell carcinoma; KICH, kidney chromophobe; KIRC, kidney renal clear cell carcinoma; KIRP, kidney renal papillary cell carcinoma; LAML, acute myeloid leukemia; LGG, brain lower grade glioma; LIHC, liver hepatocellular carcinoma; LUAD, lung adenocarcinoma; LUSC, lung squamous cell carcinoma; MESO, mesothelioma; OV, ovarian serous cystadenocarcinoma; PAAD, pancreatic adenocarcinoma; PCPG, pheochromocytoma and paraganglioma; PRAD, prostate adenocarcinoma; READ, rectum adenocarcinoma; SARC, sarcoma; SKCM, skin cutaneous melanoma; STAD, stomach adenocarcinoma; TGCT, testicular germ cell tumors; THCA, thyroid carcinoma; THYM, thymoma; UCEC, uterine corpus endometrial carcinoma; UCS, uterine carcinosarcoma; UVM, uveal melanoma.

Leukemia is a hematologic malignancy originating in the bone marrow, characterized by the abnormal proliferation of blood cells. Four main types of leukemia can be distinguished: acute lymphoblastic leukemia (ALL), acute myeloid leukemia (AML), chronic lymphocytic leukemia (CLL), and chronic myeloid leukemia (CML) [72]. Disturbances in the hDicer level have been implicated in distinct leukemia subtypes. The extremely high overexpression of *DICER1* was found in AML (in TCGA database referred to as LAML) [73, 74, 75] (Fig. 3), and reduced levels of hDicer were detected in CLL, CML, and ALL [76, 77, 78, 79]. Interestingly, in ALL, overexpression of *DICER1* has also been reported [80], which indicates that, depending on the cancer stage, the *DICER1* expression may be affected differently.

Several reports have shown the elevated levels of *DICER1* expression in samples from the bone marrow of AML patients [74, 75] and leukemia cell lines [74], confirmed on both mRNA [74, 75] and protein [74] levels. However, since no difference in overall survival of AML patients with increased *versus* decreased expression of *DICER1* in bone marrow was found [75], it was suggested that the influence of *DICER1* expression on clinical outcomes may depend on the tissue of origin [75]. Moreover, it was shown that in AML bone marrow samples, the *DICER1* gene expression was upregulated by the hematopoietic transcription factor, GATA1 [74], whose mutations are highly significant in AML patients [81]. In addition, upregulation of hDicer was found in various myelomas [82, 83] and T‐cell lymphoma [84]. Abnormal expression of the *DICER1* gene can affect the course of diseases [85, 86, 87, 88].

While the precise cause of leukemia remains unclear, genetic mutations and environmental factors, such as smoking, ionizing radiation, viral infections, and exposure to harmful chemicals, are believed to play a role in its development and progression. These factors contribute to DNA damage [72]. As mentioned above, hDicer is one of the proteins involved in damaged DNA repair [89, 90]. Knockdown of *DICER1* has been found to reduce DSB repair [91] and increase the accumulation of unscheduled R loops [57]. It is also important to mention that loss of a single allele of *DICER1* enhances tumorigenesis (e.g., by increased amounts of oncogenic *versus* tumor suppressive miRNAs) [46], while its complete loss leads to the accumulation of DNA damage and cell death [90]. Indeed, silencing of the *DICER1* gene in AML cells inhibited cell proliferation and promoted apoptosis [74].

Reduced levels of *DICER1* expression were observed in chemotherapy‐resistant CML cells [79]. In this leukemia, the rs13078 *DICER1* variant (a single nucleotide polymorphism located in the 3′‐UTR of the *DICER1* gene) was extensively examined [92]. Both the minor allele frequency and the minor homozygote genotype of rs13078 were significantly more prevalent in CML patients than in healthy controls. This variant is thought to alter the interaction of miRNAs or RNA‐binding proteins with *DICER1* transcripts, potentially affecting the hDicer protein level and global miRNA expression, which may drive CML pathogenesis [92].

Reduced hDicer levels have also been reported in CLL [76, 77]. The *DICER1* expression analysis revealed statistically significant differences between ill and healthy samples (based on RNA isolated from 30 patients and 29 controls), where the *DICER1* gene was downregulated in the patients with CLL compared to the controls [76]. Another study has demonstrated that reduced levels of hDicer in CLL are strongly associated with shorter overall survival and reduced treatment‐free survival [77]. In this context, it is also important to remember that the reduced hDicer level may impact DSB repair and R loop regulation.

A significantly reduced *DICER1* level was also observed in thyroid cancer (THCA) [93] (Fig. 3). Thyroid cancer originates from thyroid parenchymal cells and has shown a steady rise in incidence globally, although mortality rates have remained stable in recent years [72, 94]. hDicer is crucial for normal thyroid gland development, and mutations in the *DICER1* gene are associated with various thyroid abnormalities [95]. A reduced level of Dicer in thyroid cancer could exacerbate the accumulation of R loops. In addition, a study from 2021 showed that thyroid hormone receptor‐associated protein 3 (Thrap3) plays a critical role in regulating R loop resolution by interacting with DEAD‐box helicase 5 (DDX5) [96]. Since both DDX5 and hDicer can regulate R loops, it would be of great importance to understand the connections among proteins involved in R loop regulation.

The abovementioned cases highlight the importance of understanding the molecular mechanisms underlying Dicer dysregulation and its contributions to cancer biology, particularly regarding R loop dynamics and their implications for genomic stability.

## R loops, lncRNAs and cancer

The understanding of abnormal R loop formation and its impact on genome instability and cancer development is extensively discussed. The list of cancer diseases linked to the excessive R loop accumulation is growing rapidly [43]. Here, we focus on unscheduled R loops formed by lncRNAs.

It has been shown that a lncRNA HOTTIP (the HOXA transcript at the distal tip) regulates oncogene expression through topologically associating domains (TAD) formation via R loops, without causing DNA damage [72]. Mammalian chromosomes are organized within the nucleus into TADs, which support gene regulation, DNA replication, and repair [97]. TADs are dynamic structures formed by actively extruding loops that are restricted by TAD boundaries, enhancing intra‐domain interactions [98, 99, 100]. HOTTIP lncRNA is abundantly expressed in AML. The HOTTIP‐mediated TAD formation via R loops drives aberrant oncogene transcription and leukemia development [101].

Abnormal accumulation of TERRA‐mediated R loops can interfere with telomere function, potentially leading to telomere‐related dysfunction, promoting genomic instability in diseases like cancer [3]. It has also been reported that TERRA forms R loops to promote homology‐directed DNA synthesis in the alternative lengthening of telomeres (ALT) pathway [102, 103, 104]. For example, TERRA contributes to ALT by recruiting the lysine‐specific demethylase 1A (LSD1) to telomeres. The interaction between TERRA and LSD1 has been shown to promote R loop formation and enhance the activity of R loop regulatory molecules, supporting telomere maintenance in ALT pathways [102]. ALT can also be promoted by RAD51‐associated protein 1 (RAD51AP1) [103, 104]. It has been shown that TERRA R loops mediated by RAD51AP1 regulate repressive chromatin at telomeres [104]. Moreover, it has been reported that in multiple myeloma, stabilization of TERRA G‐quadruplexes induced dissociation of telomeric repeat‐binding factor 2 (TRF2) from telomeres, leading to the activation of the DNA damage response, cell cycle arrest, proliferation block, and apoptotic death [105].

## Perspectives

There are reports suggesting that R loops can serve as diagnostic biomarkers. Their unscheduled accumulation has emerged as a promising diagnostic biomarker, with the potential to stratify patients and monitor disease severity across various conditions, including immune deficiencies like Wiskott–Aldrich syndrome [106], leukemia [72], embryonal tumors with correlation to loss of *DICER1* function [107], and glioblastomas [108], also in correlation with *DICER1* expression [109, 110, 111].

Landmark studies over the past decade have shown that small extracellular vesicles (sEVs, also referred to as “exosomes” [112]) from human blood contain genomic DNA fragments reflecting the host cell genome [111]. Notably, sEV‐DNA from cancer patients accurately mirrors the mutational status of the original tumor cells, highlighting its potential as a liquid biopsy biomarker for cancer detection and monitoring metastasis [111]. In addition, recent studies have shown the existence of microvesicles containing miRNAs and lncRNAs, which may activate multiple pathways involved in tumor development when transferred to the target cells [113, 114, 115]. The microvesicle‐derived lncRNAs might as well contribute to unscheduled R loop formation. Elevated R loop levels are linked to genomic instability and tumor heterogeneity, and their presence offers prognostic value, guiding therapeutic approaches and predicting outcomes in disorders such as multiple myeloma [116] and uterine fibroids [117], particularly when analyzed alongside extracellular vesicle‐derived genetic materials [111]. This could lead to a breakthrough in diagnosing diseases by utilizing easily accessible biological samples, such as blood, for rapid and accurate testing.

## Conflict of interest

The authors declare no conflict of interest.

## Author contributions

AK‐K conceptualized the work, KW and PK wrote the draft of the manuscript. KW prepared all figures and Table 1. AK‐K revised and edited the manuscript and was responsible for its final form. All authors have read and agreed to the published version of the manuscript.

## References

[1] Santos‐Pereira JM and Aguilera A (2015) R loops: new modulators of genome dynamics and function. Nat Rev Genet 16, 583–597. 26370899 10.1038/nrg3961

[2] Lee CY , McNerney C , Ma K , Zhao W , Wang A and Myong S (2020) R‐loop induced G‐quadruplex in non‐template promotes transcription by successive R‐loop formation. Nat Commun 11, 3392. 32636376 10.1038/s41467-020-17176-7 PMC7341879

[3] Gong Y and Liu Y (2023) R‐loops at chromosome ends: from formation, regulation, and cellular consequence. Cancers (Basel) 15, 2178. 37046839 10.3390/cancers15072178 PMC10093737

[4] Masukata H and Tomizawa J (1990) A mechanism of formation of a persistent hybrid between elongating RNA and template DNA. Cell 62, 331–338. 1695550 10.1016/0092-8674(90)90370-t

[5] Drolet M and Brochu J (2019) R‐loop‐dependent replication and genomic instability in bacteria. DNA Repair 84, 102693. 31471263 10.1016/j.dnarep.2019.102693

[6] Kreuzer KN and Brister JR (2010) Initiation of bacteriophage T4 DNA replication and replication fork dynamics: a review in the virology journal series on bacteriophage T4 and its relatives. Virol J 7, 358. 21129203 10.1186/1743-422X-7-358 PMC3016281

[7] Mosig G (1987) The essential role of recombination in phage T4 growth. Annu Rev Genet 21, 347–371. 3327469 10.1146/annurev.ge.21.120187.002023

[8] Giles RE , Blanc H , Cann HM and Wallace DC (1980) Maternal inheritance of human mitochondrial DNA. Proc Natl Acad Sci USA 77, 6715–6719. 6256757 10.1073/pnas.77.11.6715 PMC350359

[9] Wai T , Ao A , Zhang X , Cyr D , Dufort D and Shoubridge EA (2010) The role of mitochondrial DNA copy number in mammalian fertility. Biol Reprod 83, 52–62. 20130269 10.1095/biolreprod.109.080887 PMC2888963

[10] Chen X , Prosser R , Simonetti S , Sadlock J , Jagiello G and Schon EA (1995) Rearranged mitochondrial genomes are present in human oocytes. Am J Hum Genet 57, 239–247. 7668249 PMC1801549

[11] Aguilera A and García‐Muse T (2012) R loops: from transcription byproducts to threats to genome stability. Mol Cell 46, 115–124. 22541554 10.1016/j.molcel.2012.04.009

[12] Yu K , Chedin F , Hsieh CL , Wilson TE and Lieber MR (2003) R‐loops at immunoglobulin class switch regions in the chromosomes of stimulated B cells. Nat Immunol 4, 442–451. 12679812 10.1038/ni919

[13] Chaudhuri J and Alt FW (2004) Class‐switch recombination: interplay of transcription, DNA deamination and DNA repair. Nat Rev Immunol 4, 541–552. 15229473 10.1038/nri1395

[14] Zarrin AA , Alt FW , Chaudhuri J , Stokes N , Kaushal D , Du Pasquier L and Tian M (2004) An evolutionarily conserved target motif for immunoglobulin class‐switch recombination. Nat Immunol 5, 1275–1281. 15531884 10.1038/ni1137

[15] Yu K and Lieber MR (2003) Nucleic acid structures and enzymes in the immunoglobulin class switch recombination mechanism. DNA Repair 2, 1163–1174. 14599739 10.1016/j.dnarep.2003.08.010

[16] Statello L , Guo CJ , Chen LL and Huarte M (2021) Author correction: gene regulation by long non‐coding RNAs and its biological functions. Nat Rev Mol Cell Biol 22, 159. 10.1038/s41580-021-00330-4 PMC8095262 33420484

[17] In S , Renck Nunes P , Valador Fernandes R and Lingner J (2025) TERRA R‐loops trigger a switch in telomere maintenance towards break‐induced replication and PrimPol‐dependent repair. *bioRxiv* 2025.01.09.632133. 10.1101/2025.01.09.632133 [PREPRINT] PMC12361433 40624280

[18] Sze S , Bhardwaj A , Fnu P , Azarm K , Mund R , Ring K and Smith S (2023) TERRA R‐loops connect and protect sister telomeres in mitosis. Cell Rep 42, 113235. 37843976 10.1016/j.celrep.2023.113235 PMC10873023

[19] Fernandes RV , Feretzaki M and Lingner J (2021) The makings of TERRA R‐loops at chromosome ends. Cell Cycle 20, 1745–1759. 34432566 10.1080/15384101.2021.1962638 PMC8525998

[20] Xu Y , Suzuki Y , Ito K and Komiyama M (2010) Telomeric repeat‐containing RNA structure in living cells. Proc Natl Acad Sci USA 107, 14579–14584. 20679250 10.1073/pnas.1001177107 PMC2930462

[21] Zeinoun B , Teixeira MT and Barascu A (2023) TERRA and Telomere maintenance in the yeast *Saccharomyces cerevisiae* . Genes (Basel) 14, 618. 36980890 10.3390/genes14030618 PMC10048448

[22] Bettin N , Oss Pegorar C and Cusanelli E (2019) The emerging roles of TERRA in telomere maintenance and genome stability. Cells 8, 246. 30875900 10.3390/cells8030246 PMC6468625

[23] Rivosecchi J , Jurikova K and Cusanelli E (2024) Telomere‐specific regulation of TERRA and its impact on telomere stability. Semin Cell Dev Biol 157, 3–23. 38088000 10.1016/j.semcdb.2023.11.001

[24] Wang C , Zhao L and Lu S (2015) Role of TERRA in the regulation of telomere length. Int J Biol Sci 11, 316–323. 25678850 10.7150/ijbs.10528 PMC4323371

[25] Bryan TM (2020) G‐quadruplexes at telomeres: friend or foe? Molecules 25, 3686. 32823549 10.3390/molecules25163686 PMC7464828

[26] Nanavaty V , Sandhu R , Jehi SE , Pandya UM and Li B (2017) Trypanosoma brucei RAP1 maintains telomere and subtelomere integrity by suppressing TERRA and telomeric RNA:DNA hybrids. Nucleic Acids Res 45, 5785–5796. 28334836 10.1093/nar/gkx184 PMC5449629

[27] Kim TK , Hemberg M , Gray JM , Costa AM , Bear DM , Wu J , Harmin DA , Laptewicz M , Barbara‐Haley K , Kuersten S *et al*. (2010) Widespread transcription at neuronal activity‐regulated enhancers. Nature 465, 182–187. 20393465 10.1038/nature09033 PMC3020079

[28] De Santa F , Barozzi I , Mietton F , Ghisletti S , Polletti S , Tusi BK , Muller H , Ragoussis J , Wei CL and Natoli G (2010) A large fraction of extragenic RNA pol II transcription sites overlap enhancers. PLoS Biol 8, e1000384. 20485488 10.1371/journal.pbio.1000384 PMC2867938

[29] Jia Q , Deng H , Wu Y , He Y and Tang F (2023) Carcinogen‐induced super‐enhancer RNA promotes nasopharyngeal carcinoma metastasis through NPM1/c‐Myc/NDRG1 axis. Am J Cancer Res 13, 3781–3798. 37693164 PMC10492133

[30] Pefanis E , Wang J , Rothschild G , Lim J , Kazadi D , Sun J , Federation A , Chao J , Elliott O , Liu ZP *et al*. (2015) RNA exosome‐regulated long non‐coding RNA transcription controls super‐enhancer activity. Cell 161, 774–789. 25957685 10.1016/j.cell.2015.04.034 PMC4428671

[31] Skourti‐Stathaki K , Kamieniarz‐Gdula K and Proudfoot NJ (2014) R‐loops induce repressive chromatin marks over mammalian gene terminators. Nature 516, 436–439. 25296254 10.1038/nature13787 PMC4272244

[32] Ginno PA , Lott PL , Christensen HC , Korf I and Chédin F (2012) R‐loop formation is a distinctive characteristic of unmethylated human CpG Island promoters. Mol Cell 45, 814–825. 22387027 10.1016/j.molcel.2012.01.017 PMC3319272

[33] Fu Y , Liu Y , Wen T , Fang J , Chen Y , Zhou Z , Gu X , Wu H , Sheng J , Xu Z *et al*. (2023) Real‐time imaging of RNA polymerase I activity in living human cells. J Cell Biol 222, e202202110. 36282216 10.1083/jcb.202202110 PMC9606689

[34] Mishra PK , Chakraborty A , Yeh E , Feng W , Bloom KS and Basrai MA (2021) R‐loops at centromeric chromatin contribute to defects in kinetochore integrity and chromosomal instability in budding yeast. Mol Biol Cell 32, 74–89. 33147102 10.1091/mbc.E20-06-0379 PMC8098821

[35] Racca C , Britton S , Hédouin S , Francastel C , Calsou P and Larminat F (2021) BRCA1 prevents R‐loop‐associated centromeric instability. Cell Death Dis 12, 896. 34599155 10.1038/s41419-021-04189-3 PMC8486751

[36] Skourti‐Stathaki K , Proudfoot NJ and Gromak N (2011) Human senataxin resolves RNA/DNA hybrids formed at transcriptional pause sites to promote Xrn2‐dependent termination. Mol Cell 42, 794–805. 21700224 10.1016/j.molcel.2011.04.026 PMC3145960

[37] Castellano‐Pozo M , García‐Muse T and Aguilera A (2012) R‐loops cause replication impairment and genome instability during meiosis. EMBO Rep 13, 923–929. 22878416 10.1038/embor.2012.119 PMC3463965

[38] Xu Y , Jiao Y , Liu C , Miao R , Liu C , Wang Y , Ma C and Liu J (2024) R‐loop and diseases: the cell cycle matters. Mol Cancer 23, 84. 38678239 10.1186/s12943-024-02000-3 PMC11055327

[39] Takeda DY and Dutta A (2005) DNA replication and progression through S phase. Oncogene 24, 2827–2843. 15838518 10.1038/sj.onc.1208616

[40] Fujiwara Y , Handel MA and Okada Y (2022) R‐loop formation in meiosis: roles in meiotic transcription‐associated DNA damage. Epigenomes 6, 26. 36135313 10.3390/epigenomes6030026 PMC9498298

[41] Liu C , Xu W , Wang L , Yang Z , Li K , Hu J , Chen Y , Zhang R , Xiao S , Liu W *et al*. (2023) Dual roles of R‐loops in the formation and processing of programmed DNA double‐strand breaks during meiosis. Cell Biosci 13, 82. 37170281 10.1186/s13578-023-01026-2 PMC10173651

[42] Bader AS and Bushell M (2020) DNA:RNA hybrids form at DNA double‐strand breaks in transcriptionally active loci. Cell Death Dis 11, 280. 32332801 10.1038/s41419-020-2464-6 PMC7181826

[43] Li F , Zafar A , Luo L , Denning AM , Gu J , Bennett A , Yuan F and Zhang Y (2023) R‐loops in genome instability and cancer. Cancers (Basel) 15, 4986. 37894353 10.3390/cancers15204986 PMC10605827

[44] Hamperl S and Cimprich KA (2014) The contribution of co‐transcriptional RNA:DNA hybrid structures to DNA damage and genome instability. DNA Repair 19, 84–94. 24746923 10.1016/j.dnarep.2014.03.023 PMC4051866

[45] Ciechanowska K , Pokornowska M and Kurzynska‐Kokorniak A (2021) Genetic insight into the domain structure and functions of dicer‐type ribonucleases. Int J Mol Sci 22, 616. 33435485 10.3390/ijms22020616 PMC7827160

[46] Kurzynska‐Kokorniak A , Koralewska N , Pokornowska M , Urbanowicz A , Tworak A , Mickiewicz A and Figlerowicz M (2015) The many faces of dicer: the complexity of the mechanisms regulating dicer gene expression and enzyme activities. Nucleic Acids Res 43, 4365–4380. 25883138 10.1093/nar/gkv328 PMC4482082

[47] Bernstein E , Caudy AA , Hammond SM and Hannon GJ (2001) Role for a bidentate ribonuclease in the initiation step of RNA interference. Nature 409, 363–366. 11201747 10.1038/35053110

[48] Fukagawa T , Nogami M , Yoshikawa M , Ikeno M , Okazaki T , Takami Y , Nakayama T and Oshimura M (2004) Dicer is essential for formation of the heterochromatin structure in vertebrate cells. Nat Cell Biol 6, 784–791. 15247924 10.1038/ncb1155

[49] Gullerova M and Proudfoot NJ (2012) Convergent transcription induces transcriptional gene silencing in fission yeast and mammalian cells. Nat Struct Mol Biol 19, 1193–1201. 23022730 10.1038/nsmb.2392 PMC3504457

[50] Verdel A , Jia S , Gerber S , Sugiyama T , Gygi S , Grewal SI and Moazed D (2004) RNAi‐mediated targeting of heterochromatin by the RITS complex. Science 303, 672–676. 14704433 10.1126/science.1093686 PMC3244756

[51] Volpe TA , Kidner C , Hall IM , Teng G , Grewal SI and Martienssen RA (2002) Regulation of heterochromatic silencing and histone H3 lysine‐9 methylation by RNAi. Science 297, 1833–1837. 12193640 10.1126/science.1074973

[52] Noma K , Sugiyama T , Cam H , Verdel A , Zofall M , Jia S , Moazed D and Grewal SI (2004) RITS acts in cis to promote RNA interference‐mediated transcriptional and post‐transcriptional silencing. Nat Genet 36, 1174–1180. 15475954 10.1038/ng1452

[53] White E , Schlackow M , Kamieniarz‐Gdula K , Proudfoot NJ and Gullerova M (2014) Human nuclear dicer restricts the deleterious accumulation of endogenous double‐stranded RNA. Nat Struct Mol Biol 21, 552–559. 24814348 10.1038/nsmb.2827 PMC4129937

[54] Burger K , Schlackow M , Potts M , Hester S , Mohammed S and Gullerova M (2017) Nuclear phosphorylated dicer processes double‐stranded RNA in response to DNA damage. J Cell Biol 216, 2373–2389. 28642363 10.1083/jcb.201612131 PMC5551710

[55] Kurzynska‐Kokorniak A , Pokornowska M , Koralewska N , Hoffmann W , Bienkowska‐Szewczyk K and Figlerowicz M (2016) Revealing a new activity of the human dicer DUF283 domain in vitro. Sci Rep 6, 23989. 27045313 10.1038/srep23989 PMC4820750

[56] Pokornowska M , Milewski MC , Ciechanowska K , Szczepanska A , Wojnicka M , Radogostowicz Z , Figlerowicz M and Kurzynska‐Kokorniak A (2020) The RNA‐RNA base pairing potential of human dicer and Ago2 proteins. Cell Mol Life Sci 77, 3231–3244. 31655860 10.1007/s00018-019-03344-6 PMC7391396

[57] Camino LP , Dutta A , Barroso S , Pérez‐Calero C , Katz JN , García‐Rubio M , Sung P , Gómez‐González B and Aguilera A (2023) DICER ribonuclease removes harmful R‐loops. Mol Cell 83, 3707–3719.e5. 37827159 10.1016/j.molcel.2023.09.021 PMC11034902

[58] Szczepanska A , Wojnicka M and Kurzynska‐Kokorniak A (2021) The significance of the DUF283 domain for the activity of human ribonuclease dicer. Int J Mol Sci 22, 8690. 34445396 10.3390/ijms22168690 PMC8395393

[59] Fragkos M , Barra V , Egger T , Bordignon B , Lemacon D , Naim V and Coquelle A (2019) Dicer prevents genome instability in response to replication stress. Oncotarget 10, 4407–4423. 31320994 10.18632/oncotarget.27034 PMC6633883

[60] Francia S , Michelini F , Saxena A , Tang D , de Hoon M , Anelli V , Mione M , Carninci P and di d'Adda Fagagna F (2012) Site‐specific DICER and DROSHA RNA products control the DNA‐damage response. Nature 488, 231–235. 22722852 10.1038/nature11179 PMC3442236

[61] Wei W , Ba Z , Gao M , Wu Y , Ma Y , Amiard S , White CI , Rendtlew Danielsen JM , Yang YG and Qi Y (2012) A role for small RNAs in DNA double‐strand break repair. Cell 149, 101–112. 22445173 10.1016/j.cell.2012.03.002

[62] Chakraborty P and Grosse F (2011) Human DHX9 helicase preferentially unwinds RNA‐containing displacement loops (R‐loops) and G‐quadruplexes. DNA Repair 10, 654–665. 21561811 10.1016/j.dnarep.2011.04.013

[63] Cristini A , Groh M , Kristiansen MS and Gromak N (2018) RNA/DNA hybrid interactome identifies DXH9 as a molecular player in transcriptional termination and R‐loop‐associated DNA damage. Cell Rep 23, 1891–1905. 29742442 10.1016/j.celrep.2018.04.025 PMC5976580

[64] Lee T and Pelletier J (2016) The biology of DHX9 and its potential as a therapeutic target. Oncotarget 7, 42716–42739. 27034008 10.18632/oncotarget.8446 PMC5173168

[65] Shivji MKK , Renaudin X , Williams ÇH and Venkitaraman AR (2018) BRCA2 regulates transcription elongation by RNA polymerase II to prevent R‐loop accumulation. Cell Rep 22, 1031–1039. 29386125 10.1016/j.celrep.2017.12.086 PMC5846855

[66] Del Baldo G , Mastronuzzi A , Cipri S , Agolini E , Matraxia M , Novelli A , Cacchione A , Serra A , Carai A , Boccuto L *et al*. (2024) The coexistence of a BRCA2 germline and a DICER1 somatic variant in two first‐degree cousins suggests their potential synergic effect. Sci Rep 14, 21435. 39271738 10.1038/s41598-024-71667-x PMC11399136

[67] Edwards DS , Maganti R , Tanksley JP , Luo J , Park JJH , Balkanska‐Sinclair E , Ling J and Floyd SR (2020) BRD4 prevents R‐loop formation and transcription‐replication conflicts by ensuring efficient transcription elongation. Cell Rep 32, 108166. 32966794 10.1016/j.celrep.2020.108166 PMC7507985

[68] Gutbrod MJ , Roche B , Steinberg JI , Lakhani AA , Chang K , Schorn AJ and Martienssen RA (2022) Dicer promotes genome stability via the bromodomain transcriptional co‐activator BRD4. Nat Commun 13, 1001. 35194019 10.1038/s41467-022-28554-8 PMC8863982

[69] Becherel OJ , Yeo AJ , Stellati A , Heng EY , Luff J , Suraweera AM , Woods R , Fleming J , Carrie D , McKinney K *et al*. (2013) Senataxin plays an essential role with DNA damage response proteins in meiotic recombination and gene silencing. PLoS Genet 9, e1003435. 23593030 10.1371/journal.pgen.1003435 PMC3623790

[70] Brickner JR , Garzon JL and Cimprich KA (2022) Walking a tightrope: the complex balancing act of R‐loops in genome stability. Mol Cell 82, 2267–2297. 35508167 10.1016/j.molcel.2022.04.014 PMC9233011

[71] Koralewska N , Szczepanska A , Ciechanowska K , Wojnicka M , Pokornowska M , Milewski MC , Gudanis D , Baranowski D , Nithin C , Bujnicki JM *et al*. (2021) RNA and DNA G‐quadruplexes bind to human dicer and inhibit its activity. Cell Mol Life Sci 78, 3709–3724. 33733306 10.1007/s00018-021-03795-w PMC8038994

[72] Lee SY , Miller KM and Kim JJ (2023) Clinical and mechanistic implications of R‐loops in human leukemias. Int J Mol Sci 24, 5966. 36983041 10.3390/ijms24065966 PMC10052022

[73] Tang Z , Kang B , Li C , Chen T and Zhang Z (2019) GEPIA2: an enhanced web server for large‐scale expression profiling and interactive analysis. Nucleic Acids Res 47, W556–W560. 31114875 10.1093/nar/gkz430 PMC6602440

[74] Bai Y , Qiu GR , Zhou F , Gong LY , Gao F and Sun KL (2013) Overexpression of DICER1 induced by the upregulation of GATA1 contributes to the proliferation and apoptosis of leukemia cells. Int J Oncol 42, 1317–1324. 23426981 10.3892/ijo.2013.1831

[75] Martin MG , Payton JE and Link DC (2009) Dicer and outcomes in patients with acute myeloid leukemia (AML). Leuk Res 33, e127. 19278725 10.1016/j.leukres.2009.02.003

[76] Farzaneh MR , Shahryari J , Safaei A , Valibeigi B , Davani SK and Tabibi N (2016) Dicer gene expression as a prognostic factor in acute lymphoblastic leukemia and chronic lymphocytic leukemia in Fars Province. Iran J Med Sci 41, 223–229. 27217607 PMC4876301

[77] Zhu DX , Fan L , Lu RN , Fang C , Shen WY , Zou ZJ , Wang YH , Zhu HY , Miao KR , Liu P *et al*. (2012) Downregulated dicer expression predicts poor prognosis in chronic lymphocytic leukemia. Cancer Sci 103, 875–881. 22320315 10.1111/j.1349-7006.2012.02234.x PMC7659218

[78] Ohtsuka M , Ling H , Doki Y , Mori M and Calin GA (2015) MicroRNA processing and human cancer. J Clin Med 4, 1651–1667. 26308063 10.3390/jcm4081651 PMC4555082

[79] Abrantes JL , Tornatore TF , Pelizzaro‐Rocha KJ , de Jesus MB , Cartaxo RT , Milani R and Ferreira‐Halder CV (2014) Crosstalk between kinases, phosphatases and miRNAs in cancer. Biochimie 107(Pt B), 167–187. 25230087 10.1016/j.biochi.2014.09.011

[80] Piroozian F , Bagheri Varkiyani H , Koolivand M , Ansari M , Afsa M , AtashAbParvar A and MalekZadeh K (2019) The impact of variations in transcription of DICER and AGO2 on exacerbation of childhood B‐cell lineage acute lymphoblastic leukaemia. Int J Exp Pathol 100, 184–191. 31090156 10.1111/iep.12316 PMC6658907

[81] Panferova A , Gaskova M , Nikitin E , Baryshev P , Timofeeva N , Kazakova A , Matveev V , Mikhailova E , Popov A , Kalinina I *et al*. (2021) GATA1 mutation analysis and molecular landscape characterization in acute myeloid leukemia with trisomy 21 in pediatric patients. Int J Lab Hematol 43, 713–723. 33386779 10.1111/ijlh.13451

[82] Shan W , Sun C , Zhou B , Guo E , Lu H , Xia M , Li K , Weng D , Lin X , Meng L *et al*. (2016) Role of dicer as a prognostic predictor for survival in cancer patients: a systematic review with a meta‐analysis. Oncotarget 7, 72672–72684. 27682871 10.18632/oncotarget.12183 PMC5341936

[83] Sarasquete ME , Gutiérrez NC , Misiewicz‐Krzeminska I , Paiva B , Chillón MC , Alcoceba M , García‐Sanz R , Hernández JM , González M and San‐Miguel JF (2011) Upregulation of dicer is more frequent in monoclonal gammopathies of undetermined significance than in multiple myeloma patients and is associated with longer survival in symptomatic myeloma patients. Haematologica 96, 468–471. 21160068 10.3324/haematol.2010.033845 PMC3046281

[84] Li X , Tian X , Zhang B and Chen J (2014) Polymorphisms in microRNA‐related genes are associated with survival of patients with T‐cell lymphoma. Oncologist 19, 243–249. 24563077 10.1634/theoncologist.2013-0370 PMC3958464

[85] Jiang C , Xu J , Zhu W , Cai Y , Wang S , Guo Y , Xu K , Geng M , Hussain N , Han Y *et al*. (2019) Abnormal expression of DICER1 leads to dysregulation of inflammatory effectors in human synoviocytes. Mediat Inflamm 2019, 6768504. 10.1155/2019/6768504 PMC6558604 31275058

[86] Caroleo AM , De Ioris MA , Boccuto L , Alessi I , Del Baldo G , Cacchione A , Agolini E , Rinelli M , Serra A , Carai A *et al*. (2020) DICER1 syndrome and cancer predisposition: from a rare pediatric tumor to lifetime risk. Front Oncol 10, 614541. 33552988 10.3389/fonc.2020.614541 PMC7859642

[87] González IA , Stewart DR , Schultz KAP , Field AP , Hill DA and Dehner LP (2022) DICER1 tumor predisposition syndrome: an evolving story initiated with the pleuropulmonary blastoma. Mod Pathol 35, 4–22. 34599283 10.1038/s41379-021-00905-8 PMC8695383

[88] Willis CL , Lucas‐Herald AK , Naotunna C , Chen SC , Davidson R , Sastry J , Murphy D , Shaikh MG and Ronghe M (2024) DICER1 syndrome and its various paediatric presentations: case series and review of the literature. EJC Paediatr Oncol 3, 100164.

[89] Bonath F , Domingo‐Prim J , Tarbier M , Friedländer MR and Visa N (2018) Next‐generation sequencing reveals two populations of damage‐induced small RNAs at endogenous DNA double‐strand breaks. Nucleic Acids Res 46, 11869–11882. 30418607 10.1093/nar/gky1107 PMC6294500

[90] Swahari V , Nakamura A and Deshmukh M (2016) The paradox of dicer in cancer. Mol Cell Oncol 3, e1155006. 27314098 10.1080/23723556.2016.1155006 PMC4909435

[91] Luan N , Mu Y , Mu J , Chen Y , Ye X , Zhou Q , Xu M , Deng Q , Hu Y , Tang Z *et al*. (2021) Dicer1 promotes colon cancer cell invasion and migration through modulation of tRF‐20‐MEJB5Y13 expression under hypoxia. Front Genet 12, 638244. 33763118 10.3389/fgene.2021.638244 PMC7982525

[92] Chavaro‐Francisco G , Hernández‐Zavala A , Bravo‐Cidro CE , Rios‐Rodriguez S , Muciño‐Sánchez M , López‐López M , Castro‐Martínez XH , Olarte‐Carrillo I , Garcia‐Laguna A , Barranco‐Lampón G *et al*. (2024) Gene variants in components of the microRNA processing pathway in chronic myeloid leukemia. Genes (Basel) 15, 1054. 39202414 10.3390/genes15081054 PMC11353722

[93] Rojo‐Pardillo M , Godefroid L , Dom G , Lefort A , Libert F , Robaye B and Maenhaut C (2024) Understanding the dosage‐dependent role of Dicer1 in thyroid tumorigenesis. Int J Mol Sci 25, 10701. 39409030 10.3390/ijms251910701 PMC11476720

[94] Boucai L , Zafereo M and Cabanillas ME (2024) Thyroid cancer: A review. JAMA 331, 425–435. 38319329 10.1001/jama.2023.26348

[95] Nosé V (2020) DICER1 gene alterations in thyroid diseases. Cancer Cytopathol 128, 688–689. 32897630 10.1002/cncy.22327

[96] Kang HJ , Eom HJ , Kim H , Myung K , Kwon HM and Choi JH (2021) Thrap3 promotes R‐loop resolution via interaction with methylated DDX5. Exp Mol Med 53, 1602–1611. 34697388 10.1038/s12276-021-00689-6 PMC8569202

[97] Szabo Q , Bantignies F and Cavalli G (2019) Principles of genome folding into topologically associating domains. Sci Adv 5, eaaw1668. 30989119 10.1126/sciadv.aaw1668 PMC6457944

[98] da Costa‐Nunes JA and Noordermeer D (2023) TADs: dynamic structures to create stable regulatory functions. Curr Opin Struct Biol 81, 102622. 37302180 10.1016/j.sbi.2023.102622

[99] Long HS , Greenaway S , Powell G , Mallon AM , Lindgren CM and Simon MM (2022) Making sense of the linear genome, gene function and TADs. Epigenetics Chromatin 15, 4. 35090532 10.1186/s13072-022-00436-9 PMC8800309

[100] Bauer M , Vidal E , Zorita E , Üresin N , Pinter SF , Filion GJ and Payer B (2021) Chromosome compartments on the inactive X guide TAD formation independently of transcription during X‐reactivation. Nat Commun 12, 3499. 34108480 10.1038/s41467-021-23610-1 PMC8190187

[101] Luo H , Zhu G , Eshelman MA , Fung TK , Lai Q , Wang F , Zeisig BB , Lesperance J , Ma X , Chen S *et al*. (2022) HOTTIP‐dependent R‐loop formation regulates CTCF boundary activity and TAD integrity in leukemia. Mol Cell 82, 833–851.e11. 35180428 10.1016/j.molcel.2022.01.014 PMC8985430

[102] Xu M , Senanayaka D , Zhao R , Chigumira T , Tripathi A , Tones J , Lackner RM , Wondisford AR , Moneysmith LN , Hirschi A *et al*. (2024) TERRA‐LSD1 phase separation promotes R‐loop formation for telomere maintenance in ALT cancer cells. Nat Commun 15, 2165. 38461301 10.1038/s41467-024-46509-z PMC10925046

[103] Yadav T , Zhang JM , Ouyang J , Leung W , Simoneau A and Zou L (2022) TERRA and RAD51AP1 promote alternative lengthening of telomeres through an R‐ to D‐loop switch. Mol Cell 82, 3985–4000.e4. 36265486 10.1016/j.molcel.2022.09.026 PMC9637728

[104] Kaminski N , Wondisford AR , Kwon Y , Lynskey ML , Bhargava R , Barroso‐Gonzalez J , Garcia‐Exposito L , He B , Xu M , Mellacheruvu D *et al*. (2022) RAD51AP1 regulates ALT‐HDR through chromatin‐directed homeostasis of TERRA. Mol Cell 82, 4001–4017.e7. 36265488 10.1016/j.molcel.2022.09.025 PMC9713952

[105] Scionti F , Juli G , Rocca R , Polerà N , Nadai M , Grillone K , Caracciolo D , Riillo C , Altomare E , Ascrizzi S *et al*. (2023) TERRA G‐quadruplex stabilization as a new therapeutic strategy for multiple myeloma. J Exp Clin Cancer Res 42, 71. 36967378 10.1186/s13046-023-02633-0 PMC10041726

[106] Wang Y , Ma B , Liu X , Gao G , Che Z , Fan M , Meng S , Zhao X , Sugimura R , Cao H *et al*. (2022) ZFP281‐BRCA2 prevents R‐loop accumulation during DNA replication. Nat Commun 13, 3493. 35715464 10.1038/s41467-022-31211-9 PMC9205938

[107] Lambo S , Gröbner SN , Rausch T , Waszak SM , Schmidt C , Gorthi A , Romero JC , Mauermann M , Brabetz S , Krausert S *et al*. (2019) The molecular landscape of ETMR at diagnosis and relapse. Nature 576, 274–280. 31802000 10.1038/s41586-019-1815-x PMC6908757

[108] Struve N , Hoffer K , Weik AS , Riepen B , Krug L , Cetin MH , Burmester J , Ott L , Liebing J , Gatzemeier F *et al*. (2022) Increased replication stress and R‐loop accumulation in EGFRvIII‐expressing glioblastoma present new therapeutic opportunities. Neurooncol Adv 4, vdab180. 35274102 10.1093/noajnl/vdab180 PMC8903237

[109] Liu YQ , Luo M , Shi Y , Guo Y , Zhang H , Yang KD , Li TR , Yang LQ , Liu TT , Huang B *et al*. (2022) Dicer deficiency impairs proliferation but potentiates anti‐tumoral effect of macrophages in glioblastoma. Oncogene 41, 3791–3803. 35764885 10.1038/s41388-022-02393-9

[110] Bronisz A , Rooj AK , Krawczyński K , Peruzzi P , Salińska E , Nakano I , Purow B , Chiocca EA and Godlewski J (2020) The nuclear DICER‐circular RNA complex drives the deregulation of the glioblastoma cell microRNAome. Sci Adv 6, eabc0221. 33328224 10.1126/sciadv.abc0221 PMC7744081

[111] Tsering T , Nadeau A , Wu T , Dickinson K and Burnier JV (2024) Extracellular vesicle‐associated DNA: ten years since its discovery in human blood. Cell Death Dis 15, 668. 39266560 10.1038/s41419-024-07003-y PMC11393322

[112] Gao Y , Qin Y , Wan C , Sun Y , Meng J , Huang J , Hu Y , Jin H and Yang K (2021) Small extracellular vesicles: A novel avenue for cancer management. Front Oncol 11, 638357. 33791224 10.3389/fonc.2021.638357 PMC8005721

[113] Kumar MA , Baba SK , Sadida HQ , Marzooqi SA , Jerobin J , Altemani FH , Algehainy N , Alanazi MA , Abou‐Samra AB , Kumar R *et al*. (2024) Extracellular vesicles as tools and targets in therapy for diseases. Signal Transduct Target Ther 9, 27. 38311623 10.1038/s41392-024-01735-1 PMC10838959

[114] Sheta M , Taha EA , Lu Y and Eguchi T (2023) Extracellular vesicles: new classification and tumor immunosuppression. Biology 12, 110. 36671802 10.3390/biology12010110 PMC9856004

[115] Piao HY , Guo S , Wang Y and Zhang J (2021) Exosome‐transmitted lncRNA PCGEM1 promotes invasive and metastasis in gastric cancer by maintaining the stability of SNAI1. Clin Transl Oncol 23, 246–256. 32519176 10.1007/s12094-020-02412-9

[116] Bradley L and Savage KI (2023) From R‐lupus to cancer': reviewing the role of R‐loops in innate immune responses. DNA Repair 131, 103581. 37832251 10.1016/j.dnarep.2023.103581

[117] Muralimanoharan S , Shamby R , Stansbury N , Schenken R , de la Pena Avalos B , Javanmardi S , Dray E , Sung P and Boyer TG (2022) Aberrant R‐loop‐induced replication stress in MED12‐mutant uterine fibroids. Sci Rep 12, 6169. 35418189 10.1038/s41598-022-10188-x PMC9008039

[118] Promonet A , Padioleau I , Liu Y , Sanz L , Biernacka A , Schmitz AL , Skrzypczak M , Sarrazin A , Mettling C , Rowicka M *et al*. (2020) Topoisomerase 1 prevents replication stress at R‐loop‐enriched transcription termination sites. Nat Commun 11, 3940. 32769985 10.1038/s41467-020-17858-2 PMC7414224

[119] de Ribeiro Almeida C , Dhir S , Dhir A , Moghaddam AE , Sattentau Q , Meinhart A and Proudfoot NJ (2018) RNA helicase DDX1 converts RNA G‐quadruplex structures into R‐loops to promote IgH class switch recombination. Mol Cell 70, 650–662.e8. 29731414 10.1016/j.molcel.2018.04.001 PMC5971202

[120] Gatti V , De Domenico S , Melino G and Peschiaroli A (2023) Senataxin and R‐loops homeostasis: multifaced implications in carcinogenesis. Cell Death Discov 9, 145. 37147318 10.1038/s41420-023-01441-x PMC10163015

[121] Bayona‐Feliu A , Herrera‐Moyano E , Badra‐Fajardo N , Galván‐Femenía I , Soler‐Oliva ME and Aguilera A (2023) The chromatin network helps prevent cancer‐associated mutagenesis at transcription‐replication conflicts. Nat Commun 14, 6890. 37898641 10.1038/s41467-023-42653-0 PMC10613258

[122] Laspata N , Kaur P , Mersaoui SY , Muoio D , Liu ZS , Bannister MH , Nguyen HD , Curry C , Pascal JM , Poirier GG *et al*. (2023) PARP1 associates with R‐loops to promote their resolution and genome stability. Nucleic Acids Res 51, 2215–2237. 36794853 10.1093/nar/gkad066 PMC10018367

[123] Fernandes RV and Lingner J (2023) The THO complex counteracts TERRA R‐loop‐mediated telomere fragility in telomerase+ cells and telomeric recombination in ALT+ cells. Nucleic Acids Res 51, 6702–6722. 37246640 10.1093/nar/gkad448 PMC10359610

[124] Cerritelli SM , Sakhuja K and Crouch RJ (2022) RNase H1, the gold standard for R‐loop detection. Methods Mol Biol 2528, 91–114. 35704187 10.1007/978-1-0716-2477-7_7

[125] Heuzé J , Kemiha S , Barthe A , Vilarrubias AT , Aouadi E , Aiello U , Libri D , Lin YL , Lengronne A , Poli J *et al*. (2023) RNase H2 degrades toxic RNA:DNA hybrids behind stalled forks to promote replication restart. EMBO J 42, e113104. 37855233 10.15252/embj.2022113104 PMC10690446

[126] Lee YJ , Lee SY , Kim S , Kim SH , Lee SH , Park S , Kim JJ , Kim DW and Kim H (2024) REXO5 promotes genomic integrity through regulating R‐loop using its exonuclease activity. Leukemia 38, 2150–2161. 39080354 10.1038/s41375-024-02362-z PMC11436357

[127] Feretzaki M , Pospisilova M , Valador Fernandes R , Lunardi T , Krejci L and Lingner J (2020) RAD51‐dependent recruitment of TERRA lncRNA to telomeres through R‐loops. Nature 587, 303–308. 33057192 10.1038/s41586-020-2815-6 PMC7116795

[128] Liu Z , Wang J , Cheng H , Ke X , Sun L , Zhang QC and Wang H‐W (2018) Cryo‐EM structure of human dicer and its complexes with a pre‐miRNA substrate. Cell 173, 1191–1203.e12. 29706542 10.1016/j.cell.2018.03.080

